# Cell Adhesion and Biofilm Development via Force-Sensitive Mechanisms: A Perspective

**DOI:** 10.1021/acsbiomaterials.5c01755

**Published:** 2025-12-24

**Authors:** Md Adnan Karim, Nooshin KianvashRad, Maurelio Cabo Jr, Samuel Chetachukwu Adegoke, Kwaniyah Tuffour, Richard Duah, Ignatius Senyo Yao Yawlui, Dennis Lajeunesse

**Affiliations:** Department of Nanoscience, 14616 Joint School of Nanoscience and Nanoengineering E Gate City Blvd, Greensboro, North Carolina 27401, United States

**Keywords:** cell adhesion, biofilm development, force-sensitive molecular switches, mechanosensation

## Abstract

Microorganisms live in environments where mechanical forces, such as fluid shear, surface tension, or pressure, shape their adhesion, biofilm formation, and maturation strategies. Microbes employ force-sensitive molecular switches embedded in surface appendages like flagella, pili, and adhesins like ALS1p or FLO11p to interpret mechanical cues. These mechanical cues trigger chemosensation or generate conformational changes in mechanosensors, thereby activating downstream signaling cascades and modulating gene expression. Ultimately, these mechanical stimuli affect microbial adhesion to surfaces, biofilm resilience, and architecture, often enhancing pathogenicity and virulence. Yet, the mechanobiological basis of these events remains underexplored. In this perspective, we discuss how bacterial and fungal systems use mechanosensation to navigate complex surfaces, underscore the challenges in monitoring real-time molecular responses to force, and explore emerging tools to reveal force-driven molecular dynamics. We highlight insights for synthetic microbiologists, materials scientists, and biomedical engineers into microbial mechanosensation and its translational potential, guiding the development of next-generation antimicrobial strategies to prevent and disrupt persistent biofilms in clinical and industrial settings.

## Graphical abstract

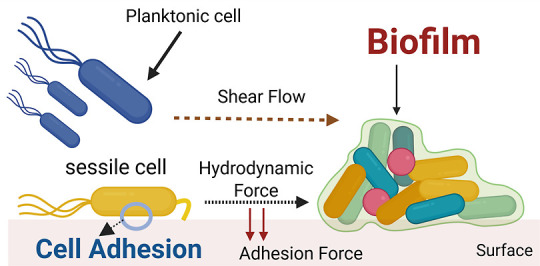

## 1 Introduction

Microbes exist in a physically turbulent world to which they have evolved many strategies to counter, adapt, and thrive. Of these adaptations, the formation of complex communities called biofilms is paramount. Biofilms are complex 3D constructions of microbial colonies that promote microbial survival by increasing resistance to antimicrobial agents, protecting cells from mechanical perturbation, and shielding microbes from host immunological defenses. These traits underscore the medical and industrial importance of understanding the mechanisms of biofilm formation and maintenance. Microbes live in dynamic environments where mechanical forces like shear, compression, and tension critically influence their adhesion and biofilm formation.^1–7^ To adapt to these physical challenges, microbes employ molecular force-sensitive switches or mechanosensors—that include specialized surface appendages, adhesion molecules including mucins and adhesins, mechanosensitive ion channels, and cytoskeletal proteins that sense surface topologies and physical stresses leading from initial reversible adhesion to irreversible attachment and eventual biofilm formation.

Many microbial mechanosensory mechanisms involve supermolecular structural appendages such as flagella and pili or specialized transmembrane channel proteins, including the large and small conductance mechanosensitive channels (MscL and MscS). These membrane-based mechanical sensing systems integrate physical force into cellular decision-making.^8–10^ These structures respond to mechanical stimuli such as shear stress, surface contact, and membrane tension through contact-based activation, which often includes conformational changes of the molecular components. These interactions convert mechanical inputs into biochemical signals, alternating metabolism, protein structure, gene expression, regulate cell motility, morphology, cell fitness, proliferation, adhesion, and biofilm formation. Sometimes, mechanical cues initiate a sequence of molecular events that lead to the production of structurally robust extracellular polymeric substances (EPS), which support the dynamic remodeling of the biofilm architecture while providing both mechanical resilience and biochemical protection. For instance, *Pseudomonas aeruginosa* (*P. aeruginosa*) modifies biofilm architecture in response to shear stress, while proteins such as Antigen 43 (Ag43) in *Escherichia coli* (*E. coli*) and clumping factor A (ClfA) in *Staphylococcus aureus* (*S. aureus*) enhance adhesion under force.^11–14^

The primary membrane/cell wall-based force detectors are surface appendages like type IV pili and flagella (Figure 1).^15,16^ Bacterial flagella are long “whip-like” filamentous macromolecular assemblies of the protein flagellin that function as a rotating motor. The primary function of flagella is motility; however, these structures also function as mechanical sensors that detect torque variations or “stall” when they encounter surfaces or differences in fluid viscosity. Mechanical sensing via flagella results in activation of signaling cascades that encourage sessile growth and changes in metabolism.^17^ Flagellar assembly is tightly coordinated with specific chemical and mechanical sensing regimes and in many cases these responses result in changes to the transcriptional state of the cell or alteration of metabolism.^18^ Flagella motility guides bacteria toward or away from a favorable environment along chemical and/or physical gradients. Recently, in the pathogenic B2 phylogroup of *E. coli,* the expression of chemosensory molecules has been shown to be governed by mechanical stresses demonstrating the interplay between these two sensory modalities and the growing complexity of mechanical stimuli in the governance of microbial behavior.^19^

**Figure 1:**
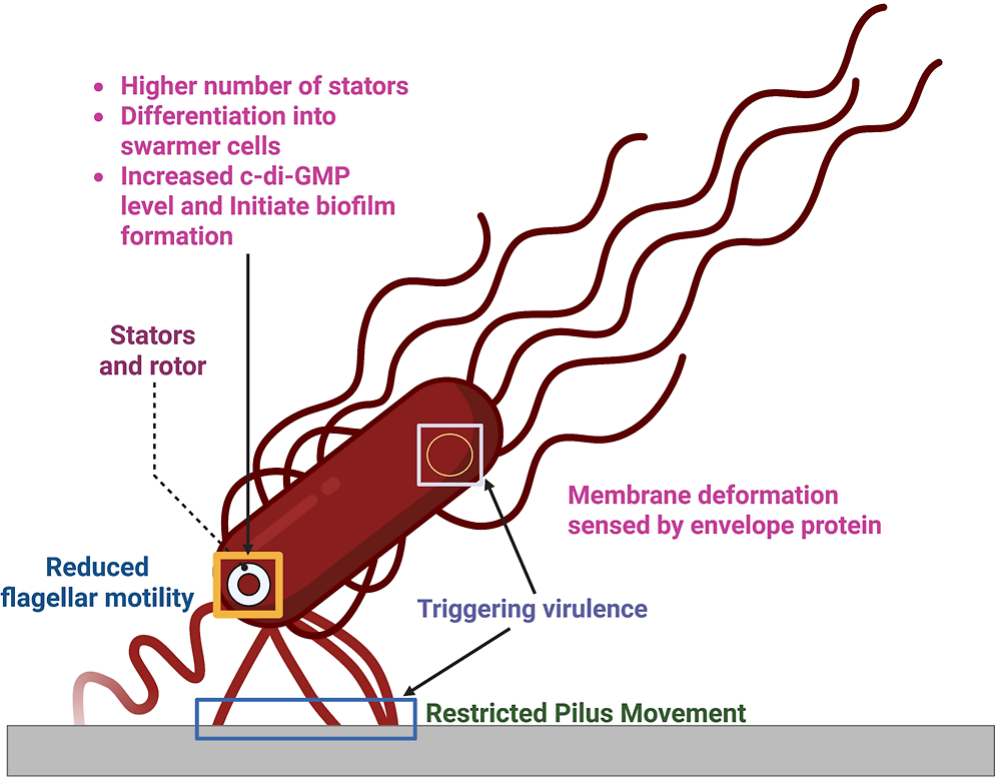
Mechanosensors in *E. coli.*Flagella, pili, and envelope proteins function as mechanosensors. Reduced flagellar motility in planktonic cells increases the number of stators in the flagellar motor, triggering swarmer cell differentiation and biofilm initiation. Restricted type IV pili movement is sensed by the Chp system, leading to upregulation of virulence. Membrane deformation is detected by the envelope protein signal transduction pathway involved in virulence. Created in BioRender. Cabo, M. (2025) https://BioRender.com/8vokrxt.

Similarly, type IV pili experience dynamic extension and retraction. Stress generated during pilus retraction and the associated “frustration”, i.e., the jamming of pili retraction, provide a mechanical signal that triggers signal transduction pathways that raise levels of intracellular secondary messengers such as cyclic AMP (cAMP) and cyclic-di-GMP (cdGMP) resulting in a twitching motility behavior and enhancement of biofilm formation.^9,20,21^ Thus, these appendages serve as mechanosensory devices and motility structures, connecting physical contact to transcriptional regulation. Although flagella and type IV pili have always been considered to have independent mechanosensing functions, recent work has shown that in *Helicobacter pylori*, flagella and type IV pili share some molecular components, enabling crosstalk between these two distinct mechanosensing systems. In these studies, homologies of the type IV pili genes^15^ PilM, PilN, and PilO are components of the *H. pylori* flagellar cage; while loss of these genes does not inhibit flagellar motility, they do prevent cells from moving into semisolid substrates, suggesting that they facilitate mechanical sensation of local viscosity. Whether such mechanisms exist in other bacterial species remains to be determined, but the sharing of molecular components demonstrates the potential for more complex responses to mechanical signals.

At the molecular level, adhesin proteins in the outer membrane detect the stiffness of the substrate and start signaling cascades to increase adherence.^22^ These systems function as both structural anchors and mechanotransducers.^9^ Adhesins change their binding characteristics in response to mechanical stress. FimH in *E. coli* is an example of a catch-bond adhesin that strengthens under tensile strain, improving colonization under flow circumstances and offering feedback on adhesion quality.^23–26^ According to Laventie & Jenal, surface detection and attachment are the main functions of adhesion-based sensors.^27^ By physically interacting with substrates, these structures implicitly detect mechanical stimuli. On the other hand, specialized membrane proteins known as mechanosensitive (MS) ion channels identify mechanical stress in the membrane or cell envelope directly without the need for substrate attachment.^28,29^ These sensors translate mechanical input into biochemical signals in response to membrane tension, cell wall strain, or osmotic shock, which is force-specific and independent of adhesion.^30–32^ It is essential for survival under osmotic and mechanical stress. Harper & Hernandez, et al. found out that in microbes, to avoid lysis during hypoosmotic shock, mechanosensitive ion channels (MscL, MscS)^33,34^ open under membrane stretch. Gerken et al. also revealed that the EnvZ/OmpR system also controls porin expression in *E. coli* by reacting to variations in turgor pressure.^35^ There is also cell wall integrity (CWI) signaling pathway in yeast that is activated by several plasma membrane mechanosensors, notably the canonical Wsc family (Wsc1, Wsc2, and Wsc3), which detect mechanical stress and cell wall strain.^36–39^ Advances in mechanobiology have further elucidated how structural proteins like actin and myosin and mechanotransduction pathways such as Rho signaling contribute to microbial adhesion and force adaptation.^40,41^

Despite significant progress, microbial mechanosensing remains underexplored compared with similar phenomena in mammalian systems. This perspective highlights microbial mechanosensors and the force-assisted mechanisms that they employ to modulate adhesion and biofilm development. Understanding these microbial strategies offers valuable opportunities for translational research and therapeutic innovation due to the evolving interface between microbiology, materials science, and mechanobiology.

## 2 How do Mechanical Forces Shape Adhesion Behavior?

Microorganisms thrive in environments where mechanical forces are constantly at play. From host tissues to industrial surfaces, interfacial forces influence how bacteria and fungi sense, respond, and ultimately decide whether they adhere or detach. Rather than passive settlers, microbes actively interpret mechanical cues to regulate adhesion mechanisms, a behavior that is central to biofilm formation and survival. Swimming bacteria, for example, are equipped with flagella and type IV pili that serve dual purposes: motility and mechanosensation (Figure 1). When flagellar movement is hindered or pili experience tension upon surface contact, these mechanical disturbances are transduced into biochemical signals that modulate adhesion and initiate biofilm development.^40,42^ This process, orchestrated by structures like flagella, type IV pili, and envelope proteins, exemplifies the tight integration of mechanical sensing with microbial behavior.^40^ A notable case is *P. aeruginosa*, where type IV pili retracts through PilT ATPase activity, pulling the bacterium closer to the surface in response to a mechanical feedback, thereby reinforcing its attachment.^43^

Microbial systems often rely on catch–slip bond dynamics to fine-tune attachment. Catch bonds strengthen under mechanical force, enabling stronger adhesion, while slip bonds weaken with increased force, facilitating detachment when needed (Figure 2C). In *E. coli*, the adhesin FimH forms robust bonds with mannose under shear flow (Figure 2B), stabilizing attachment in high-stress environments such as the urinary tract.^25^ However, when force exceeds a critical threshold, these bonds weaken—allowing the bacteria to detach and relocate to more favorable conditions.^44^ Surface proteins called adhesins also play a central role in microbial interaction with both biotic and abiotic surfaces. Shear flow activates most fungal adhesins by unfolding pseudostable protein domains that expose cross-β sequences (Figure 2A), key to amyloid formation on the cell surface.^45^ Cross-β bonding, triggered by shear stress, acts as a catch mechanism that enhances adhesion to surfaces and epithelia and promotes fungal cell aggregation during early biofilm formation.^45^ For example, yeast cells aggregate more under shear as unfolded adhesins expose cross-β sequences, forming stable bonds between cells and clustering into high-avidity nanodomains.^46–48^ *Candida albicans* cells also bind more strongly under laminar flow as shear stress unfolds adhesins like Als5, exposing amyloid-forming regions that cluster into high-avidity patches and strengthen adhesion via catch-bond-like cross-β interactions.^49–52^

**Figure 2:**
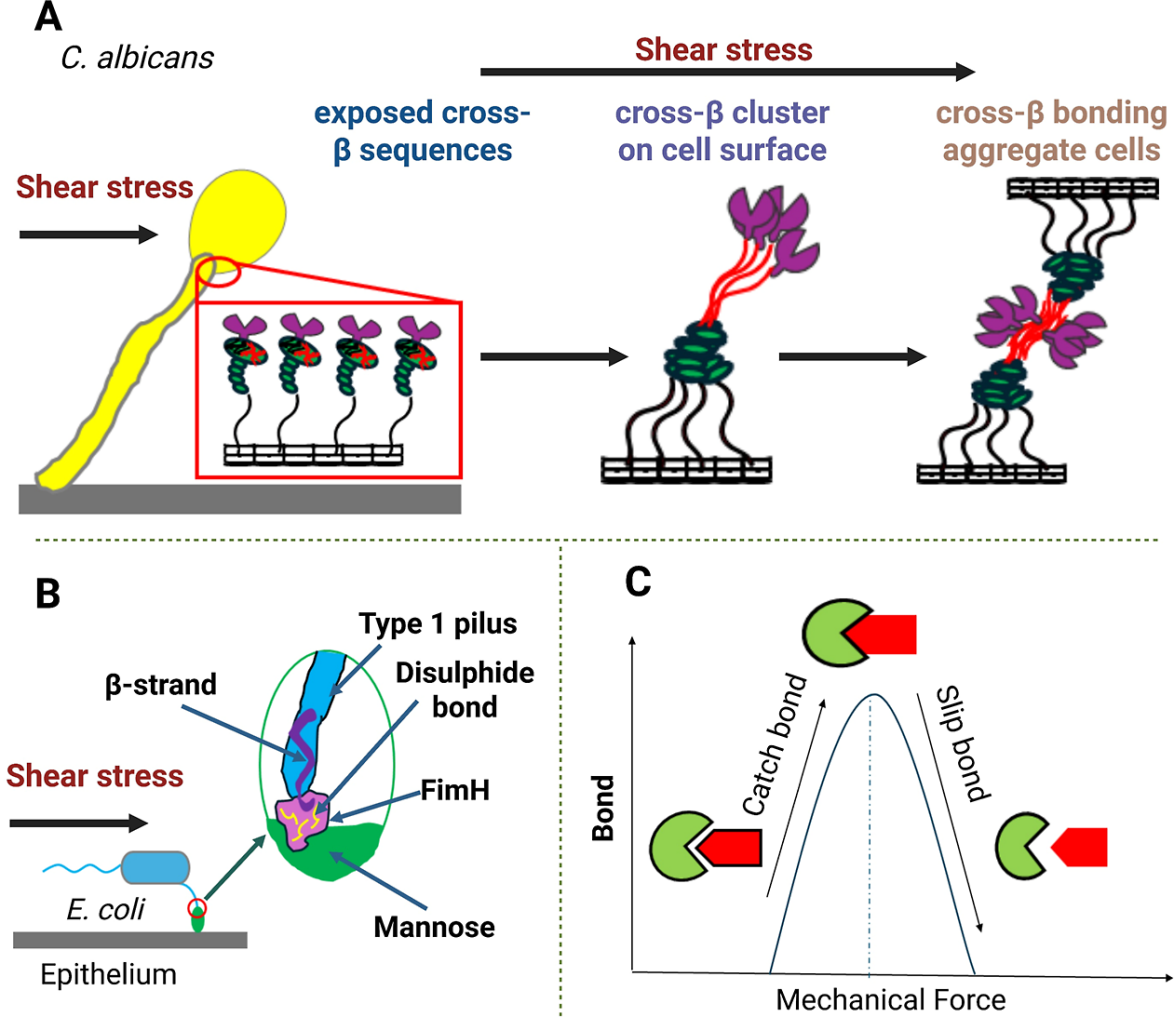
Force-induced adhesion system. (A) Shear flow unfolds Als5 on the cell surface and exposed amyloidogenic cross-β sequences. These flexible stalks strengthen and stabilize adhesion under flow by forming intermolecular cross-β cluster and cross-β bonding. (B) Shear stress induces conformational changes in *E. coli* FimH that promote β-strand complementation and disulfide bond formation to maintain the structural stability of the lectin domain. Together, these mechanisms preserve a robust mannose-binding pocket under force. (C) Catch bonds strengthen under mechanical force, enhancing cell–surface adhesion. Slip bonds initiate beyond a threshold force, when shear stress exceeds cell–surface adhesion strength, facilitating cell detachment from the surface. Created in BioRender. Cabo, M. (2025) https://BioRender.com/8vokrxt.

In addition, mechanical forces influence downstream signaling cascades. Such forces can initiate gene expression and posttranslational modifications linked to biofilm formation; for example, upon sensing surface contact via restricted flagellum rotation, *Bacillus subtilis* (Figure 3A) activates the DegS-DegU circuit to promote biofilm formation by expressing key genes like *bslA*.^53^ Planktonic *P. aeruginosa* cells use type IV pili (T4P) (Figure 3B) as force-generating appendages whose retraction generates mechanical signals used for surface sensing.^43^ Similarly, when a pilus contacts the surface and successfully retracts, this mechanical signal activates the enzyme CyaB, which increases intracellular cAMP levels. Rising cAMP enhances twitching motility, enabling efficient surface exploration.^43,54^ However, if a *P. aeruginosa* cell encounters Psl, the mannose-rich exopolysaccharide trail is deposited by other cells and the surface adhesin CdrA binds to it, creating resistance to T4P retraction. The mechanical resistance generated by this CdrA–Psl interaction triggers increased production of c-di-GMP, the second messenger that promotes the transition toward biofilm formation.^20^ Altogether, these findings underscore the pivotal role of mechanical stimuli in the microbial behavior. A single adhesive event, influenced by mechanical cues, can cascade into the growth of a mature resistant biofilm.

**Figure 3:**
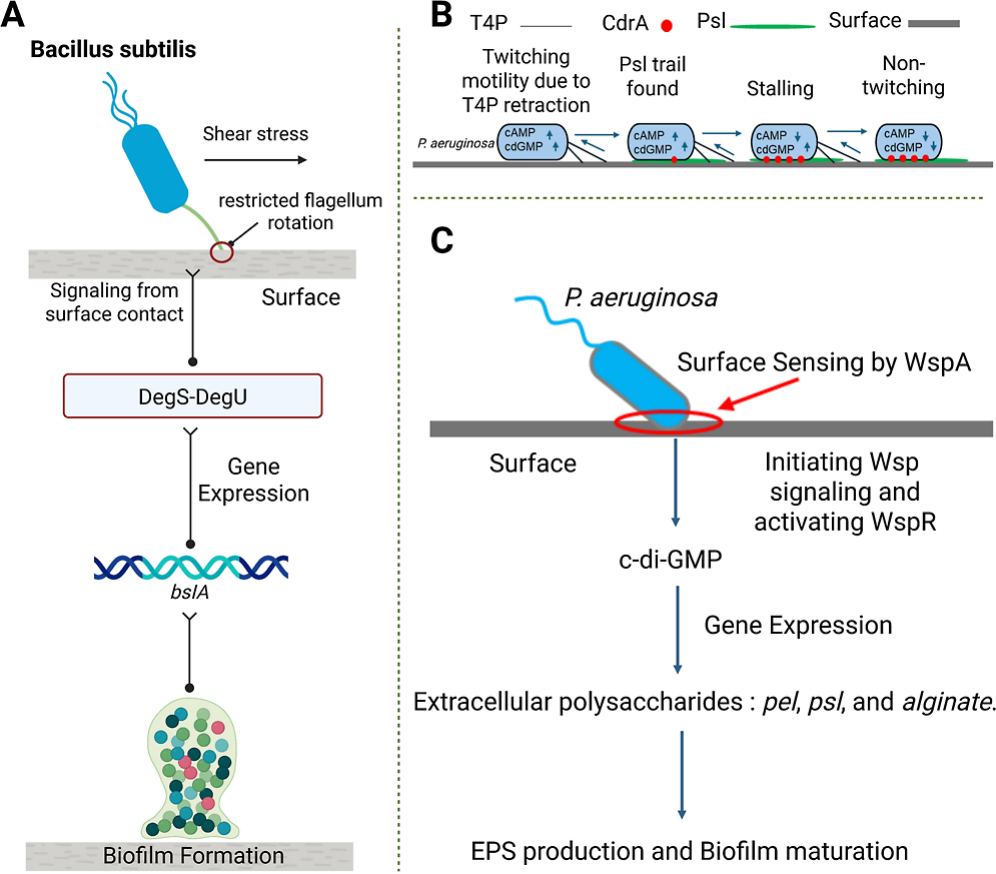
Force-triggered signal transduction pathways driving biofilm formation and maturation. (A) In *Bacillus subtilis,* restricted flagellar movement from surface activates the DegS-DegU two-component system, inducing biofilm genes such as *bsIA.* (B) *P. aeruginosa* explores surfaces using type IV pili (T4P). Successful T4P retraction generates a mechanical signal that increases intracellular cAMP, enhancing twitching motility. When the pilus encounters PsI trails deposited by preceding cells, the adhesin CdrA binds to PsI and resists retraction. This frustration in retraction increases c-di-GMP levels, driving transition toward stable adhesion and biofilm formation. (C) In *P. aeruginosa* *,* surface contact sensed by WspA activates the Wsp pathway, leading to WspR activation, which synthesizes c-di-GMP. Elevated c-di-GMP induces genes for extracellular polysaccharides (*Pel, PsI, Alginate*), producing a structurally robust matrix that supports stable microcolony formation and biofilm maturation. Created in BioRender. Cabo, M. (2025) https://BioRender.com/8vokrxt.

Table 1 summarizes key mechanosensitive molecules and their force-induced responses that regulate microbial adhesion and biofilm formation.

**Table 1:** Force-Triggered Microbial Adhesion Mechanisms

mechanosensitive molecule	species	type of force applied	force triggered adhesion mechanism	ref
type 1 pilus domains	*E. coli*	shear force	upon application of force type 1 pilus in *E. coli* forms disulfide bonds and β-strand complementation to create stable and reversible catch bonds with the host epithelium responsible for robust adhesion	^55^
type IV pili	*P. aeruginosa*	long-range constant force	long-range constant force induces conformational changes to type IV pili resulting in constant force plateaus that promote adhesion or act as a mechanical signal of surface engagement for the bacterium	^56^
PilY1	*P. aeruginosa*	mechanical force	one or more cysteine residues in the vWA domain (disulfide bonds) of the TFP tip-associated protein PilY1 undergoes conformational change during the application of force and binds to surface. Besides, PilY1 exhibits hierarchical unfolding and Ca^2+^-dependent stabilization of its C-terminal β-propeller and integrin-binding domains under applied force, revealing a force-sensitive adhesion at the single-molecule level	^57,58^
FnBPs, SdrF, SasG, SdrG	*Staphylococcus epidermidis* (*S. epidermidis*) and *S. aureus*	shear stress, loading forces	upon the application of force, conformational changes are observed in fibronectin-binding proteins (FnBPs), collagen-binding protein (SdrF), and surface protein G (SasG), which enhance adhesion. Additionally, the serine–aspartate repeat protein (SdrG) binds to fibrinogen through a ″dock, lock, and latch″ mechanism	^13,59–63^
Doc, Coh	*Ruminococcus champanellensis* (*R. champanellensis*)	shear stress	catch–slip bond mechanism detected between the Dockerin (Doc) and Cohesin (Coh) proteins. Bond becomes stronger under increased mechanical force (shear stress) and weakens when the force is reduced	^64^
ClfA	*S. aureus*	shear stress	catch-bond behavior is observed between ClfA and fibrinogen. Force induces conformational changes in ClfA, enhancing its binding to fibrinogen and promoting stronger adhesion	^13^
AlS proteins	*C. albicans*	mechanical force	upon application of mechanical force, the amyloid sequences in ALS proteins get exposed, which leads to aggregation (cis and trans) and enhanced adhesion	^49,52,65–68^
RrgB	*Streptococcus pneumoniae* (*S. pneumoniae*)	shear stress	shear stress induces conformational changes in RrgB proteins within the pilus-1 filament, exposing collagen-binding sites in the D2 and D3 domains. The D2 and D3 domains then act as a molecular hook and anchors the bacterium to the host tissue	^69^
pilus-tip adhesin Cpa	Streptococcus pyogene (S. pyogene)	shear stress	mechanical stress modulates the thioester bond in the pilus tip adhesin Cpa through a catch-bond-like behavior that prolongs the lifetime of the Cpa–ligand interaction. When the applied force exceeds a threshold, unfolding of the CnaB domain leads to thioester bond cleavage, rendering the adhesin temporarily inactive. Upon force relaxation, the protein refolds and reconstitutes the thioester bond to restore its binding ability	^70^

Defining underlying mechanisms will be critical for developing strategies to disrupt adhesion on both biotic and abiotic surfaces in medical and industrial settings—either by designing innovative surfaces that modulate force transmission or by directly targeting microbial mechanosensory pathways.

## 3 Mechanical Regulation of Biofilm Architecture and Stability

Sensing and responding to mechanical forces are survival strategies that shape the microbial behavior and the structural organization of biofilms. A striking example is cariogenic oral biofilms, where *Streptococcus mutans* uses surface-bound glucosyltransferases to rapidly synthesize glucans from dietary sugars, forming a protective EPS matrix that anchors microcolonies to tooth surfaces.^71^ This matrix serves both as an adhesive layer and as a mechanically responsive scaffold, fostering biofilm maturation and defense.

Mechanical forces, whether through fluid shear, cellular motility, or internal biofilm pressure, are powerful regulators in biofilm development. Self-imposed mechanical stress from a confined growth environment activates the rpoH stress pathway in *E. coli*, triggering matrix production in stressed regions through a feedback loop that promotes biofilm development and antibiotic tolerance.^72^ *B. subtilis* biofilms also generate internal mechanical stress through confined growth and cell proliferation, which—together with extracellular polymeric substance (EPS) secretion—builds compressive force that reinforces structural integrity, drives morphological changes like wrinkling, enables self-repair, and helps shape the biofilm.^73^ Mechanical forces contribute to both adhesion and cohesion, reinforcing biofilm integrity at multiple scales.^40^ Hwang, Klein^71^ demonstrated that late-stage *S. mutans* biofilms required 2.5× higher shear force for detachment and retained 2.4× more biomass compared to earlier stages, underscoring the biomechanical resilience biofilms develop over time. Cutting-edge techniques now allow researchers to probe these mechanical phenomena—from the nanoscopic interactions of bacterial appendages to the shear resistance of mature biofilms.^74^

The role of mechanical stimuli extends beyond physical structure—it intertwines with molecular signaling. In *P. aeruginosa* *,* the Wsp pathway is activated due to surface contact (Figure 3C), which modulates intracellular c-di-GMP levels.^75,76^ Elevated c-di-GMP then triggers EPS production and biofilm maturation.^77–80^ Intracellular messengers like cyclic-di-GMP (c-di-GMP) translate mechanical stress into behavioral switches, driving the transition from motile to sessile lifeforms.^81–83^ Both the Wsp system (Figure 3C) and the frustration in T4P retraction (Figure 3B) produce c-di-GMP. cAMP is the output of T4P retraction. c-di-GMP output from these two systems raises an important question: can the same surface stimulate both pathways? Integrin-like signaling and two-component systems (TCS) further coordinate responses to mechanical inputs, fine-tuning adhesion and matrix production.^84^ These insights reveal a compelling perspective: biofilms are not just passive microbial blankets but force-responsive living systems. Understanding these mechanobiological-based dialogues within a biofilm offers promising avenues for disrupting pathogenic biofilms and designing next-generation antimicrobial materials that target their mechanical weak points.

In Table 2, we list recent studies identifying mechanosensitive molecules and their force-triggered responses that regulate biofilm formation and maintenance.

**Table 2:** Force-Triggered Biofilm Formation and Maintenance Mechanisms

mechanosensitive molecule	species	type of force applied	force-triggered mechanism	ref
PilA	*Streptococcus agalactiae* (*S. agalactiae*)	shear stress	PilA facilitates the aggregation of bacterial cells, promoting the transition from an early-stage biofilm to a mature, stable, and robust biofilm structure	^85^
Pel and Psl	*P. aeruginosa*	shear stress	Pel and Psl exopolysaccharides dynamically remodel the biofilm matrix by balancing stability and flexibility, with Psl enhancing structural support and Pel increasing viscosity for effective spreading	^86^
CsgA	*E. coli*	shear stress	reinforce the biofilm structure by interacting with the extracellular matrix, leading to more cohesive and durable biofilms. This is achieved through the formation of amyloid fibrils, which provide a robust scaffold that supports the biofilm’s architecture and resilience	^87^
FapC	Pseudomonas	shear stress	FapC contributes to mature biofilm formation by forming amyloid fibrils that enhance the mechanical strength and stability of the biofilm matrix	^87^
GtfB^2^	*C. albicans* - *Streptococcus mutans*	shear force	GtfB helps mature biofilm formation by binding to mannans on the surface of *C. albicans*. This binding enhances the production of the glucan matrix, which is crucial for the structural integrity and stability of the biofilm, thereby promoting the coexistence of *Streptococcus mutans* and *C. albicans* within the biofilm	^88,89^
GtfB	*C. albicans*	shear force	GtfB promotes mature biofilm formation by increasing the expression of *Candida albicans* genes such as HWP1, ALS1, and ALS3	^88^

## 4 Perspectives, Challenges, and Future Direction

Microbes have evolved force-sensitive molecules and structures that allow them to sense and respond to mechanical forces—such as favorable and unfavorable surface topologies and changes in fluid viscosity—to regulate adhesion and promote biofilm formation, especially in mixed-species communities and clinical settings. Gram-negative, Gram-positive bacteria, and fungi have evolved different strategies for interpreting and utilizing mechanical force from their environment. Gram-negative bacteria such as *E. coli* and *P. aeruginosa* use a direct integration of mechanical signals to alter their metabolism. Using mechanosensor molecules such as the *E. coli* FimH or the *P. aeruginosa* PilY1 expressed on the tips of surface appendages such as flagella or pilI, mechanical signals are directly transduced into the cell. In contrast, Gram-positive bacteria evolved an indirect signaling process. Gram-negative bacteria such as *Staphylococcus epidermidis*, *S. aureus*, and *S. pyogenes* have mechanosensitive adhesins (FnBPs, SdrF, SasG, SdrG, Cpa) in their outer membrane that undergo conformational change upon force stimulation, which controls surface adhesion and triggers changes within the cell to induce biofilm. In fungal species like *Saccharomyces cerevisiae* and *C. albicans,* mechanical force unfolds specific cell wall adhesins (Flo or Als proteins), exposing their hidden cross-β sequences resulting in cellular aggregation, which facilitate adhesion and biofilm formation. Both bacterial and fungal species exhibit a catch-bonding behavior, and mechanical force is a major factor for both species to initiate adhesion and biofilm formation. In summary, Gram-negative bacteria interpret mechanical cues as environmental signals to activate surface-sensing pathways and trigger biofilm formation, whereas Gram-positive bacteria use force to mechanically strengthen their covalently anchored adhesins to resist high shear stress. Fungal species, however, rely on a different mechanism: mechanical force exposes their hidden nanodomains (cross-β sequences) in the cell membrane, promoting the formation of cross-β aggregates or amyloids that enhance adhesion and aggregation, resulting in robust biofilms. Because these force-responsive switches differ fundamentally between bacteria and fungi, strategies to control their biofilms are different. Moreover, as fungi are eukaryotes, and they undergo hyphal transitions^90^ and produce a unique extracellular matrix reinforced by force-induced amyloids,^50^ it is particularly difficult to treat a fungal biofilm formed under mechanical force.

There are single-molecule and cell-level biophysical methods to probe mechanosensitive molecules. Techniques like atomic force microscopy (AFM),^91^ optical tweezers,^92^ and magnetic tweezers^70^ enable precise application of pico-to nanonewton force to quantify force-dependent conformational changes, unfolding events, and binding kinetics. AFM applies force using cantilever deflection; optical tweezers rely on manipulation of beads through a focused laser; and magnetic tweezers apply forces through magnetic fields on functionalized beads. These techniques offer exceptional force resolution and temporal control but remain low-throughput and instrumentation-intensive and require careful calibration. Single-cell force spectroscopy (SCFS),^93^ an AFM variant, extends this capability to whole cells by attaching an intact cell to the cantilever to measure cell–cell or cell–surface adhesion and detachment forces under defined loading rates. Although highly informative for adhesion-mediated mechanosensing, SCFS is sensitive to cell-to-cell variability. Single-molecule mechanochemical sensing (SMMS)^94^ converts binding events into mechanical outputs using AFM or tweezers, achieving high temporal resolution while remaining limited by stochastic variability. FRET-based tension sensors^95^ measure piconewton loads on specific proteins through force-dependent changes in fluorophore separation in nanometer precision but require extensive calibration and computational analysis. Plasmonic tension nanosensors (PTNS)^96^ detect single-molecule forces through force-induced optical shifts in metallic nanostructures and offer high temporal sensitivity but demand complex fabrication.

At the cellular scale, traction force microscopy (TFM) and micropillar or micropost arrays map forces exerted on deformable substrates, like bead-embedded gels providing a partial view of the intracellular force generation.^91^ On the other hand, particle tracking microrheology (PTM)^97^ probes intracellular mechanics by monitoring tracer motion, revealing viscoelasticity and motor activity but without linking signals to specific biological processes. Together, these methods span piconewton molecular forces to whole-cell traction measurements, providing a comprehensive and scalable toolbox for mechanosensor characterization.

Mechanosensitive molecules remain poorly understood due to major technical challenges. A key limitation is the lack of tools to monitor molecular conformational changes in real time. *E. coli* and other model organisms have advanced the field, but they are often confined to simplified in vitro systems that do not capture the fluid shear, pressure, and mechanical dynamics biofilms face in vivo.^83,98^ For example, studies on *C. albicans* show that biofilm architecture shifts under physiological forces—yet simulating such conditions in the lab remains difficult.^99,100^ A single-molecule force spectroscopy, optical tweezers, advanced microfluidics,^101–103^ next-generation imaging tools such as force-sensing fluorophores,^104^ and super-resolution microscopy^105^ offer fresh insight into the ways microbes and their biofilms respond to mechanical challenges. New microfluidic systems mimic fluid flow within living systems, and interstitial spaces are necessary to replicate conditions that microbes and biofilms experience in medical devices and blood vessels under shear stress and flow conditions.

The implications of this work extend far beyond the lab bench. Force-sensitive molecular switches could become targets for disrupting stubborn biofilms in medical settings, from catheters to wound beds.^106–111^ Looking ahead, future research must aim to decode these by altering gene expression networks. Integrating computational models to simulate these forces—from protein-scale deformations to community-wide shear response—could significantly improve our predictions of biofilm behavior.^112^ These models will be especially useful when paired with real-world data from in vivo environments such as the lungs and gastrointestinal tract, where biofilms contribute to persistent infections like cystic fibrosis or inflammatory bowel disease. They will also inform cancer research, where similar mechanotransduction pathways guide tumor cell adhesion and migration. As we refine our understanding of microbial force sensing, we open new opportunities to design smarter biomaterials and therapeutic interventions, turning microbial mechanics into actionable science.
